# Medically refractory epilepsy comorbid with psychogenic non-epileptic seizures and intellectual disability in a patient with balanced t(14;X) translocation: a case report

**DOI:** 10.1186/s13256-026-06432-x

**Published:** 2026-07-31

**Authors:** Henry Y. Feng, Ping Li

**Affiliations:** 1 Department of Molecular and Cellular Biology, University of California, Berkeley, Berkeley, CA USA; 2 Department of Neurology, Jacobs School of Medicine and Biomedical Sciences, State University of New York at Buffalo, Buffalo, NY USA

**Keywords:** Treatment-resistant epilepsy, Deep brain stimulation, Psychogenic non-epileptic seizures, Forced normalization, X-autosome translocation, Intellectual disability, Vagus nerve stimulation, Neuromodulation

## Abstract

**Background:**

Epilepsy affects over 50 million people worldwide, and approximately 30% of cases are treatment-resistant. Neuromodulatory therapies such as vagus nerve stimulation (VNS) and deep brain stimulation (DBS) of the anterior thalamic nuclei can reduce seizure frequency in these patients but carry risks to behavior and mood. Balanced X-autosome translocations, including t(14;X), have been implicated in intellectual disability and epilepsy and represent a rare potential genetic contributor to treatment-resistant epilepsy. We report a case that illustrates both the psychiatric risks of DBS and the possible genetic basis of refractory epilepsy.

**Case presentation:**

We present the case of a white American woman in her 30 s with treatment-resistant epilepsy (TRE), psychogenic non-epileptic seizures, intellectual disability, psychosis, and a maternally inherited balanced t(14;X)(q32.3;p11.2) translocation. Her mother also carries the same translocation and has a history of refractory epilepsy, psychosis, and cutaneous telangiectasias. Despite multiple antiepileptic drugs, vagus nerve stimulation, and deep brain stimulation (DBS) of the anterior thalamic nuclei, she continues to experience daily seizures. DBS implantation led to approximately 20% seizure reduction but was complicated by worsening behavioral disturbances, consistent with reported psychiatric side effects and the phenomenon of forced normalization.

**Conclusions:**

This case highlights the complex interplay between epilepsy, psychiatric comorbidities, and neurostimulation, as well as the potential genetic underpinnings of TRE. The association of balanced X-autosome translocations with intellectual disability and epilepsy suggests pathogenic disruption of neurodevelopmental genes influencing inhibitory signaling. Awareness of psychiatric risks in DBS recipients and consideration of genetic etiologies are essential for optimizing management strategies in refractory epilepsy.

## Background

Epilepsy is one of the most common neurological disorders worldwide, affecting over 50 million individuals, with approximately 30% of cases consisting of treatment-resistant epilepsy (TRE). Patients with TRE face disproportionate burdens due to the medically intractable nature of the epilepsy, including frequent seizures, medication toxicity, cognitive decline, and psychiatric comorbidities that together lead to significant disability and reduced quality of life. In recent years, neuromodulatory therapies such as vagus nerve stimulation (VNS) and deep brain stimulation (DBS) of the anterior thalamic nuclei (ANT) have provided additional treatment avenues for individuals, where pharmacotherapy fails to adequately manage symptoms. While DBS has demonstrated efficacy in reducing seizure frequency, its effects on behavior and mood remain a concern given the connectivity of the thalamus with limbic and prefrontal circuits.

Epilepsy also has strong genetic underpinnings, particularly in early-onset, intractable forms. Balanced X-autosome translocations, such as t(14;X) in this case, have been implicated in intellectual disability, neurodevelopmental delay, and epilepsy syndromes. Although these rearrangements do not necessarily produce overt chromosomal imbalances, pathogenicity can arise through gene disruption at breakpoints or altered epigenetic regulation.

## Case presentation

This case report presents a 37-year-old white American female with a lifelong history of epilepsy, psychogenic non-epileptic seizures (PNES), intellectual disability (ID), psychosis, and a maternally inherited balanced [t(14;X)(q32.3;p11.2)] translocation. The patient was seen at a follow-up appointment for a recent hospitalization after she reportedly bumped her head on the bed at her group home. Group home staff additionally reported her continuing to have seizures, although no generalized tonic–clonic seizures were observed.

Her family history is significant: her mother also carries the same apparently balanced t(14;X)(q32.3;p11.2) translocation and has a history of refractory epilepsy, psychosis, and cutaneous telangiectasias affecting the legs, arms, and face. This maternal inheritance pattern supports a genetic basis for the patient’s clinical phenotype.

Her history of epilepsy began at 6 years of age after experiencing a grand-mal seizure. Through her childhood and into early adolescence, her epilepsy worsened, and her seizures grew in intensity and frequency. In an attempt to treat her epilepsy, she was placed on multiple antiepileptic drugs to no avail and eventually had a vagus nerve stimulation (VNS) device implanted at 12 years of age. Despite polytherapy with an array of antiepileptic drugs and the VNS device, she continued to experience daily seizures and by the time she began high school, she required a wheelchair for mobility due to her atonic seizures. By adulthood, her epilepsy had progressed to multiple seizures per day with accompanying psychosis, with each episode lasting 30–60 min.

Multiple seizure types have been documented. The first seizure type is characterized on EEG by bifrontal and central spike-and-wave discharges evolving to rhythmic theta activity with secondary generalization; the corresponding semiology consists of automotor onset, behavioral arrest, and bilateral hand automatisms. The second seizure type shows a similar EEG pattern of bifrontal and central spike-and-wave evolving to rhythmic theta with secondary generalization, but the semiology is different—sleep arousal followed by behavioral arrest. Atonic seizures, which necessitated wheelchair use during adolescence, were also documented. No slow spike-and-wave complexes were identified on EEG, and no formal epilepsy syndrome diagnosis (e.g., Lennox–Gastaut syndrome) was established. The patient also exhibited paroxysmal events including body shaking and holding her legs up without any accompanying epileptiform activity on EEG, consistent with PNES.

Neuroimaging with MRI of the brain, performed in August 2021, was reported as normal, with no structural lesion identified to account for the seizure disorder. A PET scan prior to DBS implantation showed subtle hypometabolism in the left parietal cortex, as well as hypermetabolism of the left thalamus relative to the right. This asymmetry in thalamic and cerebral metabolism is suspected to be due to seizure activity around the time of FDG injection. A MEG scan prior to DBS implantation identified interictal dipoles in the right frontal and left frontoparietal regions, with ictal dipoles in the left inferior frontal region and frontoparietal operculum.

Long-term video EEG monitoring showed interictal background slowing with bifrontal generalized spike-wave complexes and left parietal sharp waves. Multiple ictal patterns were recorded with variable corresponding clinical effects. Generalized spike-and-wave patterns corresponded with behavioral arrest. Paroxysmal fast activity followed by rhythmic slowing with sharp waves in the left central parietal region and left central rhythmic theta–delta activity did not result in any clinical signs.

At 34 years of age, she had a deep brain stimulation (DBS) device implanted, targeting the bilateral anterior nuclei of the thalamus. Prior to DBS, she was experiencing daily seizures including convulsive generalized tonic–clonic seizures (GTCs) occurring a few times per year. However, while she initially tolerated the DBS implantation procedure well, increasing the stimulation current from 3.0 mA to 3.5 mA resulted in behavioral deterioration, prompting reprogramming to lower stimulation parameters. After returning to reduced settings (2.5 mA, cycling 1 min on/5 min off), the patient continues to exhibit behavioral abnormalities, particularly increased aggressive behaviors, although her behavioral deterioration may be attributed in part to her pre-existing history of anxiety disorders.

The patient has now been followed for approximately 3.5 year post-DBS implantation. Following DBS, she has experienced approximately 20% reduction in overall seizure frequency. Convulsive GTC seizures are now rare, occurring only 1–2 times per year and with shorter duration. However, she continues to have daily seizures of other types. The current DBS settings have been maintained at 2.5 mA with a cycling protocol of 1 min on/5 min off to balance seizure control against neuropsychiatric side effects.

The patient’s epilepsy is currently managed by the DBS and VNS devices, as well as five antiepileptic drugs (Clonazepam 0.5 mg BID, Cannabidiol 6.8 mg BID, Phenobarbital 97.2 mg BID, Lacosamide 200 mg BID and 50 mg QHS, and Zonisamide 300 mg BID). Despite the extensive range of therapies implemented to manage the patient's epilepsy, seizures continue to be observed daily, although GTC seizures have become rare.

## Discussion

DBS of the bilateral anterior nuclei of the thalamus (ANT) has emerged as an effective device therapy used in patients, where pharmacotherapy fails to adequately manage seizures. However, given the connections between the anterior thalamus with limbic and prefrontal circuits, ANT–DBS has been demonstrated to have potential side effects on behavior and mood. It is well-recognized that epilepsy and psychiatric disorders are bidirectionally associated, with up to one-third of people with epilepsy experiencing a lifetime psychiatric illness [6].

The introduction of neurostimulation further increases the risk of serious symptoms, particularly in patients with pre-existing neuropsychiatric disorders. Clinical trials have documented notable psychiatric side effects in a subset of DBS patients. In the SANTE trial of ANT–DBS, 14–15% of patients reported new or worsened depression during the blinded stimulation phase, compared to only ~ 2% in the sham group [2]. Over long-term follow-up, the prevalence of depressive mood symptoms rose to 37% of patients [8], and about 12% reported suicidal ideation, although most of those who reported suicidal ideation had prior histories of depression. Anxiety symptoms and psychotic symptoms have additionally been observed in some patients during chronic stimulation.

Fortunately, these psychiatric effects are usually reversible with adjustments to stimulation parameters, such as reducing stimulation current or duty cycle duration. For example, one report described two DBS recipients with prior histories of depression who developed acute depressive episodes immediately after certain programming settings, and two others with no prior history of psychosis who gradually developed paranoid anxiety [3], in all cases, changing the DBS settings led to resolution of the psychiatric symptoms. This reversibility suggests that DBS itself, likely the intensity and/or location of stimulation, may contribute to the psychiatric irregularities.

It is also worth noting that cognitive and behavioral effects of VNS, including memory effects and mood changes, have been reported in the literature, though these are generally mild and often considered secondary to improved seizure control [1]. In our patient, the relative contributions of longstanding VNS therapy versus the more recently introduced DBS to her behavioral profile cannot be fully disentangled, and this remains a clinical consideration.

In the context of our patient, the worsening behavioral disturbances after DBS despite a partial reduction in seizures is consistent with these reported phenomena. It is possible that her history of psychosis and intellectual disability may have predisposed her to such complications. It is noteworthy that improving seizure control can sometimes unmask or exacerbate psychiatric symptoms, a phenomenon known as forced normalization: the emergence of psychosis or behavioral disorder when epileptic activity is suppressed in a previously uncontrolled patient. This has been described with various therapies, including pharmacological treatments, VNS, and DBS. It is thought to reflect neurochemical shifts when ictal control is suddenly established. One case report describes a man with intellectual disability and medically intractable epilepsy who became delusional after his seizures abruptly ceased post-VNS implantation [5].

In our patient, a similar mechanism could explain why reducing her seizure burden via DBS coincided with heightened psychotic and behavioral issues, in addition to the inherent risks of neuropsychiatric side effects as a result of thalamic stimulation. Awareness of this possibility is critical in determining adequate treatment plans and calls for close psychiatric monitoring of DBS patients and a careful titration of stimulation to maintain a delicate balance that controls seizures without inducing intolerable mood or behavioral changes.

Treatment-resistant epilepsy (TRE) in pediatric patients is increasingly understood as a manifestation of underlying genetic and neurodevelopmental disorders, especially when conventional imaging fails to reveal a focal structural lesion. Literature has linked early-onset, intractable epilepsy to chromosomal abnormalities, single-gene mutations, and disorders of neuronal development, particularly those affecting GABAergic interneurons and the excitatory/inhibitory (E/I) balance in the maturing brain [7].

Balanced chromosomal translocations involving the X chromosome and autosomes, particularly the t(14;X), have been associated with intellectual disability, developmental delay, and various epilepsy syndromes [4,7]. Although such reciprocally balanced translocations do not result in any gross loss or duplication of genetic material, their pathogenicity could arise from gene disruption at breakpoints or aberrant gene regulation due to altered chromatin structure or skewed X-inactivation. Disruptions involving FGF13 and PHF8, two X-linked genes critical for neurodevelopment, have been implicated in prior t(14,X) cases with phenotypes including generalized epilepsy with febrile seizures plus (GEFS +) and intellectual disability [4,7]. Notably, FGF13 plays a role in neuronal excitability and the function of voltage-gated sodium channels, while PHF8 is a histone demethylase involved in X-linked intellectual disability and cleft lip/palate [4].

A particularly relevant study [7] described a family with a t(X,14)(q27;q21) translocation disrupting FGF13, in which affected males exhibited febrile seizures in childhood progressing to refractory temporal lobe epilepsy in adolescence and adulthood. Animal models of Fgf13 haploinsufficiency showed decreased inhibitory synaptic input and heightened neuronal excitability, supporting the theory that deficits in GABAergic signaling underlie the clinical phenotype [7]. This mechanism aligns with the broader hypothesis that impaired interneuron development or migration during critical periods of brain maturation can lead to a lasting E/I imbalance, predisposing individuals to seizures that become increasingly resistant to treatment over time.

Although the precise impact of a 14;X translocation varies with the genes affected, the clinical trajectory observed in many of these cases, including early febrile seizures followed by medically refractory focal epilepsy, supports a model in which subtle widespread disruption of inhibitory networks contributes to epileptogenesis. This hypothesized mechanism provides strong implications for diagnosis and management, as traditional neuroimaging and EEG findings may underestimate the extent of diffuse network dysfunction. In such cases, genome sequencing can be instrumental in identifying causal mutations and informing prognosis, potential targeted therapies, or eligibility for clinical trials. Given these findings, it is reasonable to hypothesize that the epilepsy observed in the present case may stem from a similar genetic disruption affecting early inhibitory circuit formation. While confirmatory genomic data are not yet available for this patient, the clinical features and resistance to therapy are consistent with previously described translocation-linked developmental epilepsies.

## Conclusions

In summary, this case highlights several key points. Deep brain stimulation (DBS) of the anterior thalamus can reduce seizure frequency in medically refractory epilepsy but is associated with risks of psychiatric and behavioral complications, particularly in patients with pre-existing psychiatric or neurodevelopmental disorders, making close psychiatric monitoring and careful programming essential. Clinicians should also be aware of the phenomenon of forced normalization, in which improved seizure control may unmask or exacerbate psychiatric symptoms, complicating management. In addition, balanced chromosomal translocations such as t(14;X) may disrupt critical neurodevelopmental genes, contributing to intellectual disability, psychiatric comorbidity, and pharmacoresistant epilepsy. Finally, in patients with early-onset treatment-resistant epilepsy without clear structural lesions, genetic testing should be pursued, as it can help identify underlying mechanisms of the epilepsy, inform prognosis, and guide consideration of emerging targeted therapies or clinical trial enrollment.

## Data Availability

All data generated or analyzed during this study are included in this published article.
